# Clinical Manifestations of Coronavirus Disease 2019

**DOI:** 10.31662/jmaj.2021-0013

**Published:** 2021-04-02

**Authors:** Satoshi Kutsuna

**Affiliations:** 1 Disease Control and Prevention Center, National Center for Global Health and Medicine, Tokyo, Japan

**Keywords:** coronavirus disease 2019, severe acute respiratory syndrome coronavirus 2, clinical manifestation

## Abstract

Coronavirus disease 2019 is an infection caused by severe acute respiratory syndrome coronavirus 2. Many symptomatic patients have influenza-like symptoms such as fever, respiratory symptoms (cough, sore throat, and nasal discharge), headache, and malaise. In some cases, oxygen is required within a week of onset, and in more severe cases, the patient is admitted to the intensive care unit after around 10 days of onset. In the COVIREGI-JP registry of hospitalized patients with coronavirus disease 2019, patients with renal dysfunction, liver disease, obesity, hyperlipidemia, hypertension, and diabetes tended to be more severely ill after hospitalization than patients without comorbidities. It has also become clear that symptoms can persist even after the acute phase has passed.

## Clinical Manifestations

The incubation period of coronavirus disease 2019 is ≤ 14 days, and most cases develop approximately 5 days after exposure ^(1), (2)^.

**Figure 1.**
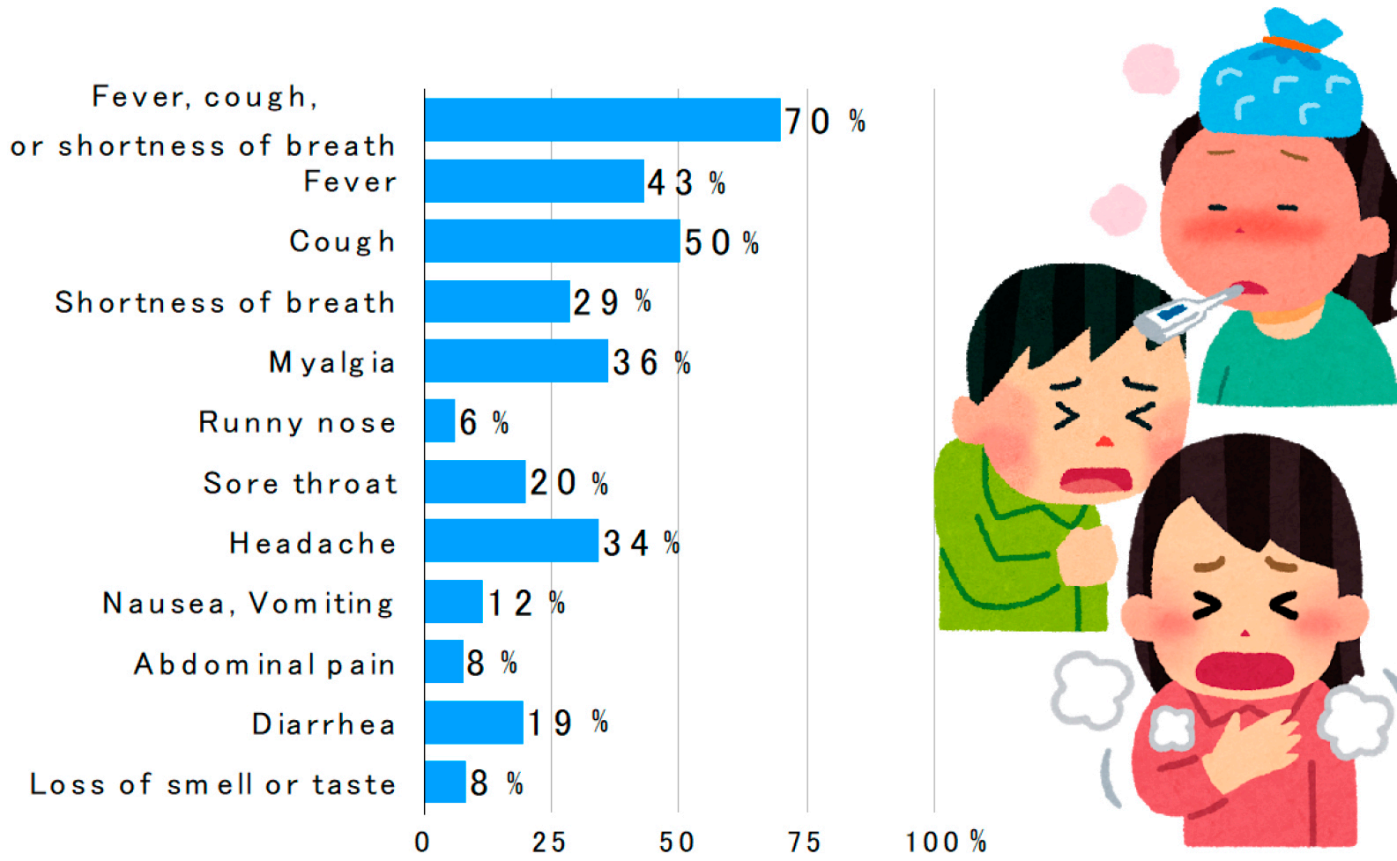
Frequency of symptoms of coronavirus disease 2019 prepared by the author based on Reference ^(4)^.

Many symptomatic patients have influenza-like symptoms such as fever, respiratory symptoms (cough, sore throat, and nasal discharge), headache, and malaise ^(2), (3)^. The frequency of clinical symptoms in 370,000 people diagnosed in the United States is shown in Figure 1 ^(4)^.

The frequency of gastrointestinal symptoms such as diarrhea and vomiting, nausea/vomiting, and abdominal pain was 13%, 10%, and 9%, respectively, in the meta-analysis ^(5)^, which is considered lower than that of SARS and MERS.

In a meta-analysis of 10 studies, the frequency of olfactory and gustatory disturbances was 52% and 44%, respectively ^(6)^. The presence of olfactory and gustatory disturbances, in addition to influenza-like symptoms, may indicate coronavirus disease 2019.

**Figure 2.**
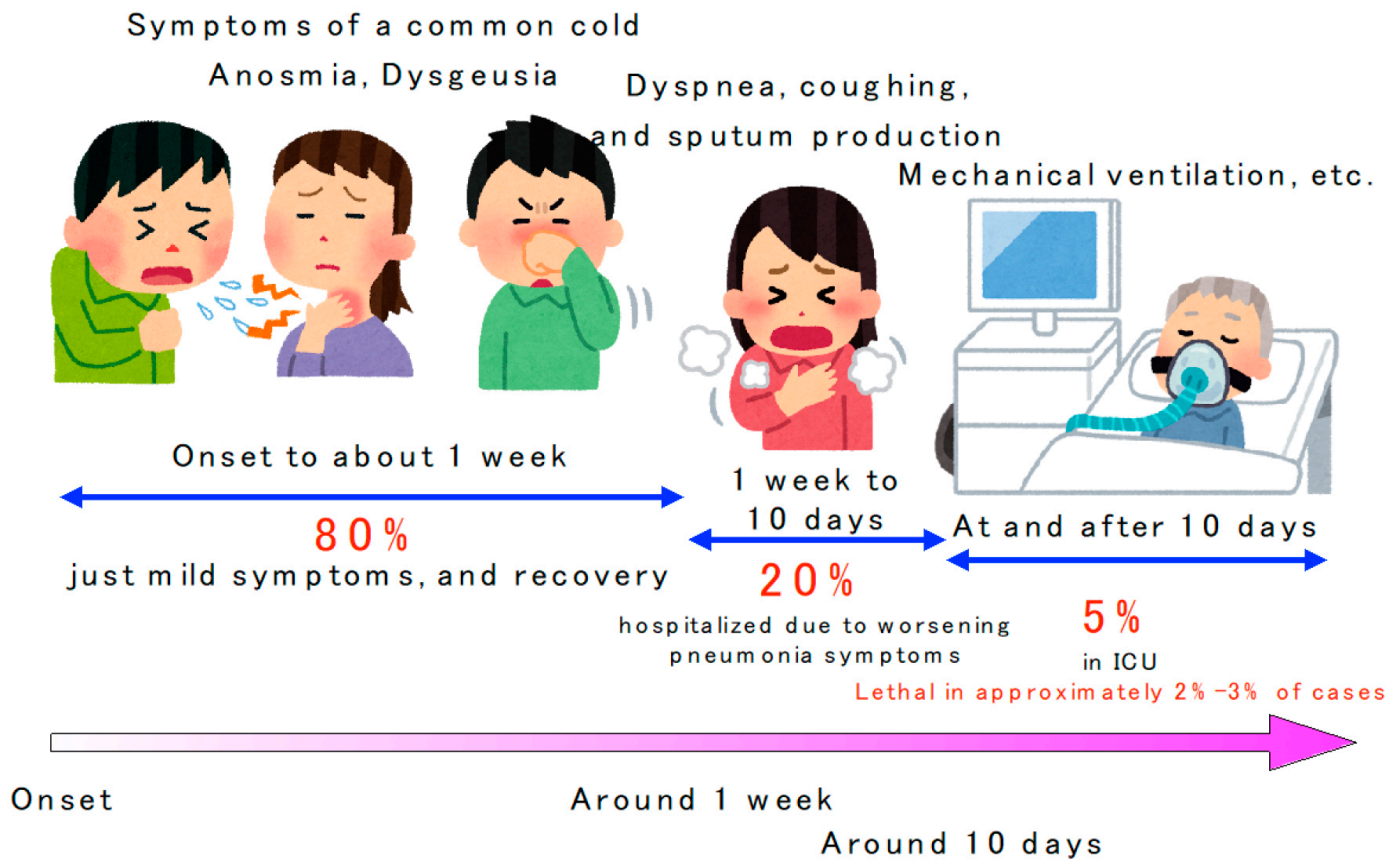
Typical course of Coronavirus disease 2019 (prepared by the author).

In some cases, oxygen is required within a week of onset, and in more severe cases, the patient is admitted to the intensive care unit after 10 days (Figure 2) ^(7)^. According to the data of 44,672 patients in China, 81% had mild disease (no or mild pneumonia), 14% had severe disease (dyspnea, hypoxemia, and pneumonia image occupying > 50% of lung area within 24-48 h), and 5% had the most severe disease (respiratory failure, shock, and multiple organ failure) ^(8)^.

According to an analysis of 2636 cases from COVIREGI-JP ^(9)^, a registry of hospitalized patients with coronavirus disease 2019 in Japan, the median age of hospitalized patients was 56 years (interquartile range: 40-71 years), and more than half of the cases were male (58.9%, 1542/2619). The median time to admission was 7 days, the median length of stay was 15 days, and the mortality rate was 7.5%. Of the 2625 admissions, 62% were mild cases that did not require oxygen, 30% were moderate cases that required oxygen, and 9% were severe cases.

## Risk Factors for Serious Illness

In the COVIREGI-JP registry of hospitalized patients with coronavirus disease 2019, patients with renal dysfunction, liver disease, obesity, hyperlipidemia, hypertension, and diabetes tended to be more severely ill after hospitalization than patients without comorbidities. In addition, compared with patients without comorbidities, patients with cardiac disease, chronic lung disease, cerebrovascular disease, and renal dysfunction tended to die at a higher rate, suggesting that the factors that lead to severe illness and death may be different. Table 1 shows the diseases and backgrounds that have been known to be risk factors for severe disease (Table 1).

**Table 1.** Risk Factors for Severe Diseases.

Risk factors for serious illness	Factors that have not yet been fully evaluated
Elderly people over 65 years old ^(9)^	
Patients with cancer ^(10)^	
Chronic obstructive pulmonary disease ^(11)^	
Chronic kidney disease ^(12)^	
Type 2 diabetes ^(13)^	Asthma ^(18)^
Hypertension ^(14)^	Use of steroids ^(19)^ or biologics ^(20)^ HIV infection (especially CD4 < 200/μL) ^(21)^
Dyslipidemia ^(9)^	Pregnancy ^(22), (23)^
Cardiovascular disease ^(15)^	
Obesity (BMI over 30) ^(16)^	
Smoking ^(14)^	
Immunodeficiency after solid organ transplantation ^(17)^	

## Symptoms That Persist in the Subacute to Chronic Phases

It has also become clear that symptoms can persist even after the acute phase has passed.

According to a report from Italy ^(24)^, 87.4% of patients still complained of some symptoms after recovering from the coronavirus disease 2019 (an average of 2 months after onset), with fatigue and respiratory distress being the most frequent symptoms. Other symptoms include arthralgia, chest pain, cough, olfactory disturbances, dry eyes and mouth, rhinitis, conjunctival hyperemia, taste disturbances, headache, phlegm, loss of appetite, sore throat, dizziness, myalgia, and diarrhea. Thirty-two percent of the patients had one or two symptoms, and 55% had three or more symptoms.

From France ^(25)^, symptoms such as hair loss, memory loss, sleep disturbance, and difficulty concentrating were also reported as sequelae. Of the 120 recovering patients who responded to a telephone interview approximately 110 days after the onset of the coronavirus disease 2019, 55%, 42%, 34%, 31%, 28%, and 24% reported fatigue, difficulty breathing, memory loss, sleep disturbance, difficulty concentrating, and hair loss, respectively. Of the 120 recovering patients who responded to the telephone interview, 55% complained of fatigue and 42%, 34%, 31%, 28% and 20% had difficulty breathing, memory problems, difficulty sleeping, difficulty concentrating, and hair loss, respectively. Symptoms such as memory impairment, poor concentration, and hair loss are sometimes seen as sequelae of infectious diseases such as Ebola and severe febrile thrombocytopenia syndrome, but they seem to be rare in the coronavirus disease 2019. Of those who were working before the coronavirus disease 2019, 69.1% had returned to work 110 days after the onset.

In a similar report from Japan, 63 people who had recovered from coronavirus disease 2019 were interviewed by phone and found that, they still had difficulty smelling (19.4%) and breathing (17.5%), lethargy (15.9%), coughing (7.9%), and dysgeusia (4.8%) 60 days after onset. Respiratory distress (11.1%), olfactory disturbance (9.7%), lethargy (9.5%), cough (6.3%), and dysgeusia (1.7%) persisted even after 120 days, and 24% had symptoms of hair loss that were not present in the acute phase ^(26)^.

**Figure 3.**
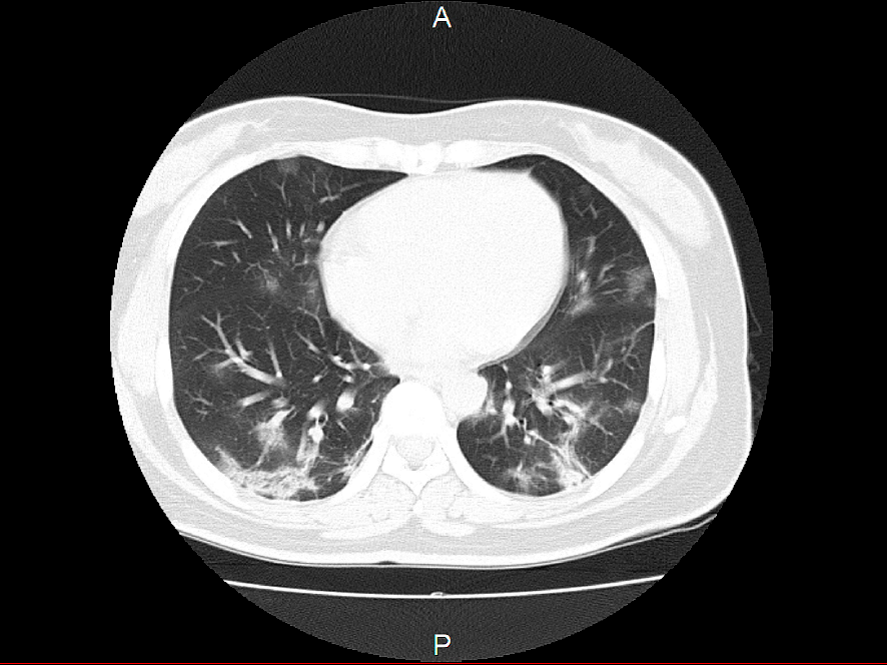
Chest CT image of a patient with coronavirus disease 2019 (a case diagnosed by the author).

Chest imaging findings are characterized by bilateral obliterating infiltrative and frosted shadows (Figure 3). In some cases, even if there is a pneumonia image on chest CT, chest X-ray cannot determine pneumonia. In a report from China, pneumonia was detected in 86.2% of chest CT images, whereas only 59.1% of chest X-rays showed pneumonia ^(2)^. Although it is not possible to simplify the results because of the differences in some images taken, chest X-rays may miss pneumonia in 20%-30% of cases. In cases with high pretest probability, such as those with contact history, a chest CT scan should be considered even if no pneumonia image is seen on chest X-ray. The pneumonia image spreads from the onset of the disease to the rest of the body, but even in asymptomatic infected patients, a chest CT scan may show a pneumonia image ^(27)^. It is a characteristic of this disease that a marked pneumonia image may be observed even in asymptomatic infected patients with no fever or respiratory symptoms.

## Article Information

### Conflicts of Interest

The author received joint research funding from Quiagen and Takeda Pharmaceutical Company Limited.
